# 阿伐曲泊帕转换治疗TPO受体激动剂难治性再生障碍性贫血疗效及安全性

**DOI:** 10.3760/cma.j.issn.0253-2727.2022.11.007

**Published:** 2022-11

**Authors:** 丽萍 井, 慧慧 樊, 文睿 杨, 园 李, 建平 李, 莉 张, 洋 李, 康 周, 佑桢 熊, 蕾 叶, 广新 彭, 洋 杨, 馨 赵, 凤奎 张

**Affiliations:** 中国医学科学院血液病医院（中国医学科学院血液学研究所），实验血液学国家重点实验室，国家血液系统疾病临床医学研究中心，细胞生态海河实验室，天津 300020 State Key Laboratory of Experimental Hematology, National Clinical Research Center for Blood Diseases, Haihe Laboratory of Cell Ecosystem, Institute of Hematology & Blood Diseases Hospital, Chinese Academy of Medical Sciences & Peking Union Medical College, Tianjin 300020, China

**Keywords:** 再生障碍性贫血，重型, TPO受体激动剂, 转换治疗, Aplastic anemia, severe, Thrombopoietin receptor agonist, Switch

## Abstract

**目的:**

评估阿伐曲泊帕（APAG）转换治疗在曾应用免疫抑制治疗（IST）联合TPO受体激动剂（TPO-RA）无效或TPO-RA不耐受的再生障碍性贫血（AA）患者中的近期疗效及安全性。

**方法:**

回顾性分析2021年1月至2021年12月中国医学科学院血液病医院（中国医学科学院血液学研究所）IST联合TPO-RA（艾曲泊帕/海曲泊帕）治疗无效或TPO-RA不耐受的16例AA患者，接受APAG转换治疗的疗效及安全性。

**结果:**

16例AA患者中，中位年龄54（14～68）岁，男、女各8例。IST联合TPO-RA治疗无效患者10例（难治组），转换APAG中位治疗6（6～10）个月，7例（70％）获得三系血液学反应（HR）［完全治疗反应（CR）4例、良好治疗反应（GPR）1例、部分治疗反应（PR）2例］，均在APAG治疗3个月时开始起效；TPO-RA不耐受患者6例（不耐受组），APAG中位治疗6（2～8）个月，4例（67％）获得HR（GPR 3例、PR 1例），其中APAG治疗3个月时2例获得PR，6个月时4例患者均获得HR。APAG转换治疗过程中，未发生APAG相关2级以上不良事件，无血栓事件发生，治疗6个月时复查骨髓病理未见纤维组织增生，无一例患者因不良事件停药。

**结论:**

对于TPO-RA难治或不耐受的AA患者，APAG可作为较好的转换治疗选择。

## Abstract (en)

**Objective:**

Short-term efficacy and safety of afatrombopag conversion therapy in patients with aplastic anemia（AA）who were previously ineffectively treated with intense immunosuppressive therapy（IST）combined with TPO receptor Agonist（TPO-RA）or who were unable to tolerate the side effects of TPO-RA.

**Methods:**

Analysis of patients with severe aplastic anemia（SAA）treated in Institute of Hematology & Blood Diseases Hospital, Chinese Academy of Medical Sciences & Peking Union Medical College from January 2021 to December 2021 who received IST combined with TPO-RA（eltrombopag/hatrombopag）for at least 6 months, but was ineffective for at least 3 months or patients who cannot continue to use TPO-RA due to side effects, and switched from TPO-RA to avatrombopag（APAG）, and treated for at least 6 months. This study analyzed its short-term efficacy and evaluated its safety.

**Results:**

The median age was 54（14–68）years old among the 16 patients with AA（8 male and eight female）. A total of ten patients（refractory group）who did not respond to IST combined with TPO-RA were converted to APAG median therapy for 6（6–10）months. Only seven patients（70％）obtained trilineage HR [four cases of complete treatment response（CTR）, one case of good treatment response（GPR）, and two cases of partial treatment response（PR）], all of which began to take effect at 3 months of APAG treatment. There were six patients with TPO-RA intolerance, and APAG was treated for 6（2 to 8）months. About four patients（67％）received HR（three GPR cases and one PR case）, of which two patients received PR at 3 months and four patients received HR at 6 months of APAG treatment. No APAG-related grade 2 or more adverse events occurred during the APAG therapy, no thrombotic events occurred, no fibrologic tissue hyperplasia was found in the bone marrow pathology reexamination at 6 months of treatment, and none of the patients discontinued the drug due to adverse events.

**Conclusion:**

APAG may be a better switching treatment option for patients with AA who are refractory or are intolerant to TPO-RA.

抗胸腺细胞球蛋白（ATG）联合环孢素A（CsA）的免疫抑制治疗（IST）是无条件接受HLA相合造血干细胞移植（HSCT）重型再生障碍性贫血（SAA）患者的一线治疗方案，血液学反应（HR）率为60％～80％ [1]–[3]，残存造血干细胞（HSC）较少被认为是疗效不佳的重要原因之一[4]。血小板生成素（TPO）及其受体激动剂（TPO-RA）与造血干/祖细胞膜受体结合可促进HSC增殖分化及多能造血祖细胞扩增，同时兼具免疫调节、诱导免疫耐受的作用[5]。重组人TPO（rhTPO）或TPO-RA艾曲泊帕（EPAG）联合IST可明显提高初治SAA患者HR率，改善血液学反应质量[6]–[8]，40％～45％的难治复发性SAA/极重型再生障碍性贫血（VSAA）患者也可获得血液学改善[9]。然而，仍有部分患者TPO-RA治疗无效或不能耐受其不良反应。新型TPO-RA阿伐曲泊帕（APAG）作用于TPO受体跨膜区，较EPAG作用效应更强且无明显肝脏毒性[10]，在我国已被批准用于肝功能异常血小板减少及难治性免疫性血小板减少的治疗，用于SAA的报道较少。本研究我们总结分析IST联合rhTPO和（或）TPO-RA［EPAG或海曲泊帕（HPAG）］治疗无效或不能耐受其不良反应的SAA患者，探索APAG转换治疗的疗效及安全性，现报道如下。

## 病例与方法

一、病例

自2021年1月至12月于中国医学科学院血液病医院贫血诊疗中心住院诊断为输血依赖非重型AA（TD-NSAA）或SAA/VSAA，接受IST联合rhTPO/TPO-RA未能获得HR或因TPO-RA不良反应无法耐受，而转换为新型TPO-RA APAG的患者。纳入本研究患者均不适于或拒绝进行异基因HSCT。AA诊断参照国际粒细胞减少与AA研究组1987年标准[11]，分型标准参照文献[12]；TD-NSAA分型诊断标准为外周血ANC<0.5×10^9^/L或PLT<20×10^9^/L或HGB<70 g/L。本研究符合赫尔辛基宣言原则及GCP规范，获得我院伦理委员会批准（批件：IIT2021008-EC-1），APAG转换治疗均征得患者本人或法定监护人知情同意并签署知情同意书。

二、APAG转换前治疗方案

1. IST方案：①ATG联合CsA：猪抗淋巴细胞免疫球蛋白（p-ATG，武汉生物制品所）20 mg·kg^−1^·d^−1^，连续应用5 d；CsA 3～5 mg·kg^−1^·d^−1^起始，分为2次服用，根据血药浓度调整剂量，维持谷浓度150～250 µg/L。②CsA单用：剂量与给药方法同前。③他克莫司（FK506）：肾功能不全患者接受FK506 0.1 mg·kg^−1^·d^−1^，分2次口服，根据血药浓度调整剂量，维持谷浓度6～10 µg/L。

2. rhTPO/TPO-RA：①rhTPO 15 000 U/d，皮下注射；②EPAG 50～75 mg/d，空腹口服；③HPAG 15 mg/d，空腹口服。

三、APAG转换及剂量调整

对于IST治疗至少6个月且rhTPO/TPO-RA治疗至少3个月未达HR（难治组），或rhTPO/TPO-RA不耐受（不耐受组）的患者，停用原rhTPO/TPO-RA并转换为APAG 40 mg/d口服，至少应用6个月；CsA或FK506继续应用并监测药物浓度。

若APAG治疗患者获得HR，PLT快速上升且>400×10^9^/L，则停药观察，直至PLT<100×10^9^/L再以20 mg/d继续治疗；若PLT>200×10^9^/L持续2周或>100×10^9^/L持续2个月，则APAG减量至20 mg/d；若仍然PLT>200×10^9^/L持续2周或>100×10^9^/L持续2个月，APAG剂量调整为20 mg隔日1次；若仍然PLT>200×10^9^/L持续2周或>100×10^9^/L持续2个月，则停药。

四、疗效标准及不良事件标准

难治性SAA疗效标准：血小板反应（HI-P）定义为PLT较基线增加≥20×10^9^/L或治疗前依赖血小板输注（PLT<20×10^9^/L）的患者至少连续8周不需输注血小板（PLT≥20×10^9^/L）；中性粒细胞反应（HI-N）定义为ANC（不使用G-CSF）较基线值增加一倍及以上或增加>0.5×10^9^/L；血红蛋白反应（HI-E）定义为HGB水平较基线增加≥15 g/L，或依赖红细胞输注（HGB<70 g/L）的患者，与入组前8周相比，连续8周红细胞输注量减少4个单位及以上。三系应答定义为同时获得HI-P、HI-N、HI-E，三系应答的反应质量同初治SAA标准。

SAA/VSAA疗效标准：①完全治疗反应（CR）：HGB>100 g/L、PLT>100×10^9^/L及ANC>1.5×10^9^/L。②良好治疗反应（GPR）：HGB>80 g/L、PLT>50×10^9^/L及ANC>1.0×10^9^/L。③部分治疗反应（PR）：脱离血制品输注依赖，血液学检查好转，不再符合SAA标准，但血常规未达到GPR标准。④无治疗反应（NR）：患者未脱离血制品输注支持治疗和（或）血液学检查仍符合SAA标准。TD-NSAA疗效判定标准：①CR：同上；②GPR：同上；③PR：至少一系达正常水平或倍增，或初始HGB<60 g/L经治疗后其水平至少升高30 g/L，或初始ANC<0.5×10^9^/L经治疗后其水平至少升高0.5×10^9^/L，或初始PLT<20×10^9^/L经治疗后其水平至少升高20×10^9^/L；④NR：血常规下降或是未达上述PR标准。

开始APAG转换治疗后3个月内死亡定义为早期死亡，纳入治疗相关不良反应及疗效评价分析。总生存时间定义为自开始APAG转换治疗至患者死亡或随访结束的时间。转换治疗后3、6个月对患者进行复查，通过外周血和骨髓细胞形态学及组织活检、PNH克隆、脏器功能、免疫指标等评估血液学反应和不良反应。不良事件的判断和定义根据《常见不良反应事件评价标准（CTCAE）》5.0版本[13]进行评价。

五、统计学处理

使用R 4.0.2和Graphpad Prism 8.0.2软件进行统计学分析和图表绘制。采用描述性统计展示患者的临床特征、实验室参数及疗效，连续变量使用中位数（范围）表示，分类变量采用频数（构成比）表示。

## 结果

一、患者一般情况

共纳入16例患者，中位年龄54（14～68）岁，男、女各8例。其中IST联合rhTPO/TPO-RA无效（难治组）患者10例，rhTPO/TPO-RA不耐受（不耐受组）患者6例。开始IST时VSAA 4例、SAA 9例、TD-NSAA 3例。

13例患者接受pATG+CsA治疗，3例因不能耐受ATG仅单用CsA，其中1例因CsA的肾功能损伤不能耐受，调整为FK506治疗。5例伴有PNH克隆，无溶血相关血液学和生化学证据。2例伴有克隆性染色体异常，分别为47,XX,+8［2/20］及45,X,−Y［10/20］（表1）。

**表1:** 16例接受阿伐曲泊帕转换治疗再生障碍性贫血患者一般临床资料

例号	性别	年龄	诊断	IST方案	转换前rhTPO/TPO-RA方案	初治结局	转换前血常规			染色体核型	PNH克隆（%）
							ANC（×10^9^/L）	HGB（g/L）	PLT（×10^9^/L）		
1	男	52	TD-NSAA	CsA	EPAG	难治	0.69	96	49	正常	1.0
2	女	68	SAA	FK506	TPO、EPAG	难治	0.38	101	1	47,XX,+8[2/20]	13.0
3	男	20	SAA	pATG+CsA	TPO、EPAG	难治	1.30	88	27	正常	1.2
4	女	51	SAA	pATG+CsA	EPAG	难治	1.66	71	35	正常	16.2
5	男	55	TD-NSAA	pATG+CsA	EPAG	难治	1.22	51	24	正常	0
6	男	55	SAA	CsA	EPAG	难治	2.00	65	12	45,X,−Y[10/20]	0
7	女	20	SAA	pATG+CsA	EPAG	难治	0.95	41	9	正常	9.8
8	女	54	VSAA	pATG+CsA	EPAG	难治	1.20	85	18	正常	0
9	男	46	SAA	pATG+CsA	TPO、EPAG	难治	1.67	44	18	正常	0
10	女	57	SAA	pATG+CsA	TPO、EPAG	难治	2.79	65	17	正常	0
11	男	14	SAA	pATG+CsA	EPAG	不耐受	0.87	57	17	正常	0
12	女	22	VSAA	pATG+CsA	TPO、EPAG	不耐受	0.49	62	39	正常	0
13	男	61	TD-NSAA	pATG+CsA	TPO、EPAG	不耐受	1.61	74	3	正常	0
14	女	45	VSAA	pATG+CsA	HPAG	不耐受	0	73	6	正常	0
15	女	55	SAA	pATG+CsA	TPO、EPAG	不耐受	0.67	70	5	正常	0
16	男	56	VSAA	pATG+CsA	TPO、EPAG	不耐受	0.08	56	9	正常	0

注 TD-NSAA：输血依赖非重型再生障碍性贫血；SAA：重型障碍性贫血；VSAA：极重型再生障碍性贫血；IST：免疫抑制治疗；CsA：环孢素A；FK506：他克莫司；pATG：猪抗淋巴细胞球蛋白；rhTPO：重组人血小板生成素；TPO-RA：血小板生成素受体激动剂；PNH：阵发性睡眠性血红蛋白尿

10例难治组患者，转换治疗前IST中位治疗时间为8.5（6～40）个月，rhTPO/TPO-RA中位治疗时间为3.5（3～10）个月。6例不耐受组患者，接受IST中位治疗时间为3（3～10）个月，rhTPO/TPO-RA中位治疗时间为3（2～3）个月。6例患者均出现肝功能异常，评估与正在使用的rhTPO/TPO-RA有关，不适于继续应用。2～3级ALT和（或）AST升高5例，2～3级总胆红素（TBIL）升高4例，其中3例为2级ALT及TBIL均升高。

二、疗效评估

难治组10例患者APAG中位治疗6（6～10）个月，7例（70％）获得三系HR（CR 4例、GPR 1例、PR 2例），均在APAG治疗3个月时开始起效；不耐受组6例患者APAG中位治疗6（2～8）个月，4例（67％）获得HR（3例为GPR，1例为PR），其中APAG治疗3个月时2例获得PR，6个月时4例患者均获得HR。HR的动态评估见图1。截止末次随访，2例患者已服用APAG 10个月，均达CR，现CsA继续维持原剂量，APAG缓慢减量过程中。

**图1:**
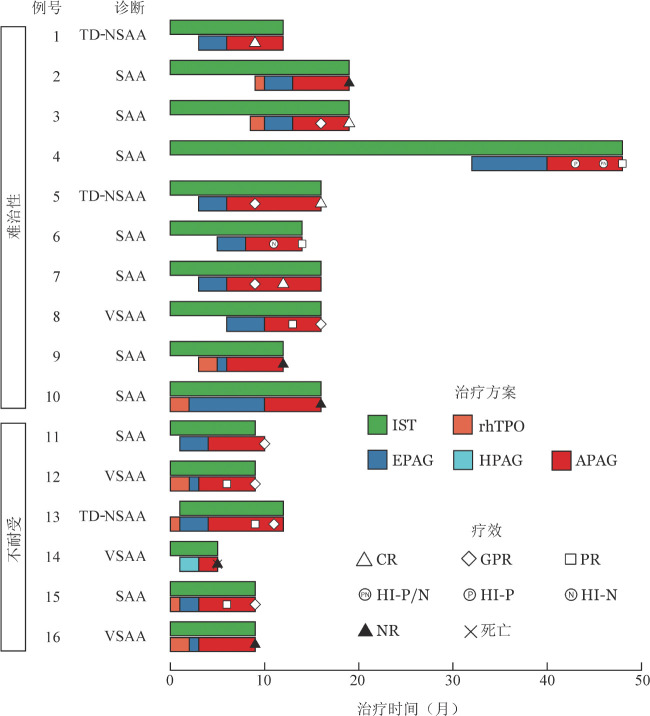
16例接受阿伐曲泊帕转换治疗再生障碍性贫血患者治疗过程及疗效泳道图 注 TD-NSAA：输血依赖非重型再生障碍性贫血；SAA：重型障碍性贫血；VSAA：极重型再生障碍性贫血；IST：免疫抑制治疗；rhTPO：重组人血小板生成素；EPAG：艾曲泊帕；HPAG：海曲泊帕；APAG：阿伐曲泊帕；CR：完全治疗反应；GPR：良好治疗反应；PR：部分治疗反应；HI-P：血小板反应；HI-N：中性粒细胞反应；NR：无治疗反应

11例APAG转换治疗后获得HR患者，外周血细胞计数多在转换治疗后1个月即开始明显改善，至转换治疗6个月过程中血常规变化见图2，ANC中位数由1.05（0.49～1.61）×10^9^/L提升至2.9（1.9～4.2）×10^9^/L；PLT中位数由20（1～49）×10^9^/L提升至96（30～231）×10^9^/L；HGB中位数由66（41～96）g/L提升至102（76～140）g/L。

**图2:**
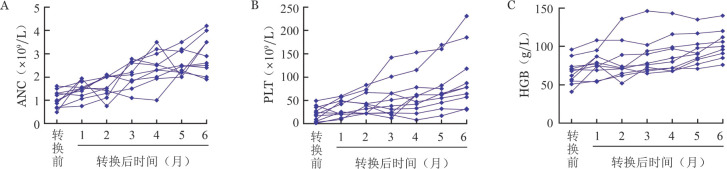
11例阿伐曲泊帕转换治疗有效再生障碍性贫血患者转换治疗前后ANC（A）、PLT（B）及HGB（C）变化

三、不良反应

16例患者APAG转换治疗过程中，未发生胃肠道及鼻咽部不适，均耐受良好。6例因肝功能异常不耐受原TPO-RA的患者，除外1例治疗后2个月死亡未随访治疗后6个月肝功能指标，余5例APAG转换治疗后肝脏功能异常未加重，而逐渐恢复，患者无明显消化系统症状发生（表2）。所有患者随访期内未发生APAG相关2级以上不良事件，无血栓事件发生，治疗6个月时复查骨髓病理未见纤维组织增生。

**表2:** 5例肝功能异常再生障碍性贫血患者阿伐曲泊帕转换治疗前后肝功能指标变化［*M*（范围）］

时间	ALT（U/L）	AST（U/L）	TBIL（µmol/L）	DBIL（µmol/L）	IBIL（µmol/L）
治疗前	52（28~356）	20（10~418）	32（10~56）	20（3~45）	15（5~25）
治疗后6个月	24（15~48）	15（12~45）	11（5~20）	5（2~10）	5（3~7）

注 ALT：丙氨酸转氨酶；AST：天冬氨酸转氨酶；TBIL：总肌红素；DBIL：直接胆红素；IBIL：间接胆红素

四、转归

1例不耐受TPO-RA的VSAA患者（例14）转换为APAG治疗2个月时因颅内出血早期死亡，疗效评价为NR。余均活存随访中，无一例患者发生骨髓增生异常综合征（MDS）及急性髓系白血病（AML）转化。

5例APAG转换治疗前伴有PNH克隆患者中，APAG治疗6个月时，1例粒细胞PNH克隆由基线的16.2％降至6.3％，1例PNH克隆由基线的9.8％升至19.6％。该2例患者均未有发作性溶血的血液学和生化学证据；另3例PNH克隆大小在APAG治疗后未有明显变化，未有新发PNH克隆和溶血PNH发生。

1例伴随+8克隆性染色体异常患者，APAG转化治疗血液学反应为NR；另1例−Y克隆性染色体异常患者获得PR，该2例患者APAG转化治疗6个月时异常克隆均持续存在。1例（例8）患者于APAG治疗3个月后新发克隆性染色体异常46,XX, t（X;4）（p11.4;q21）［4/20］，疗效评估为GPR。

## 讨论

ATG+CsA治疗无效的AA患者加用EPAG[9]、罗米司亭（ROM）[14]或HPAG[15]的血液学改善率为40％～83％。随着EPAG的一线应用临床试验获得成功[6]–[7]，采用IST联合TPO-RA方案的病例增多，TPO-RA难治性AA逐渐成为SAA治疗的新挑战。本研究结果显示，对于TPO-RA难治性SAA患者，若患者无条件或拒绝接受HSCT挽救治疗，采用TPO-RA转换治疗或为一可行替代治疗策略。

不同的TPO-RA制剂的作用靶点和效能并不完全一致。体外研究表明，TPO-RA以浓度依赖的方式刺激表达人c-MPL的Ba/F3细胞增殖，促进人脐血CD34^+^细胞向巨核细胞分化，APAG的50％效应浓度（EC50）是EPAG的3.65～6.84倍，明显强于后者[16]。针对原发免疫性血小板减少症（ITP）的临床研究结果表明，APAG可有效升高ITP患者PLT，起效速度及疗效持续时间明显优于EPAG[17]。慢性ITP的患者EPAG治疗无效，转换为APAG 50％～80％的患者获得疗效[18]。本组10例TPO-RA难治性AA患者中，除1例应用EPAG 2个月外，余9例均使用至少3个月，限于亚裔人群本品药代动力学特征及不良反应考量，使用剂量多为75 mg/d，推算其剂量效应远不如40 mg/d APAG（相当于EPAG 150～275 mg/d）。转换为APAG治疗后7例获得HR，我们认为这可能与使用APAG较EPAG有更强的TPO-RA作用效能相关。

rhTPO是与内源性TPO结构相同的生物制剂，是否同样能与AA患者内源性IFN-γ形成异二聚体，影响下游信号传导不详，尽管用于ITP疗效明确[19]，回顾性小样本研究提示IST联合rhTPO也可提高AA患者的早期疗效，改善血液学反应质量[20]–[21]，但缺少与其他TPO-RA的对照研究。APAG作用于TPO受体跨膜区，靶点与内源性TPO和rhTPO作用于TPO受体胞外区明显不同。韩国学者曾报告IST联合EPAG治疗无效的AA患者，转换为作用于TPO受体胞外区的ROM治疗，10例患者中7例（70％）获得至少1系HR[14]。但本研究中4例TPO-RA难治性AA患者曾短暂（1～2个月）使用rhTPO和至少2个月EPAG无效，转换为APAG治疗，仅1例获CR，其余3例均无效。提示TPO-RA难治性AA药物转换治疗HR的获得可能更取决于TPO-RA的作用强度，而非变更作用靶点。

IST联合EPAG一线治疗SAA的前瞻性队列研究[6]和随机对照研究[7]结果表明，EPAG可加快血液学反应的获得，明显改善血液学反应质量，并提高患者HR率；常见不良反应为1～2级血清间接胆红素升高，不需调整剂量，3～4级ALT升高发生率为18％、AST升高发生率为12％、TBIL升高发生率18％，部分患者须减量或暂时停用EPAG[7]。HPAG与EPAG分子结构相似，药效学和安全性更优[15]，Ⅱ期临床试验治疗难治性SAA，部分患者同样表现明显转氨酶升高。本组6例对EPAG和HPAG治疗不能耐受的初治AA患者，均表现为肝功能异常。APAG转换治疗后4例（67％）获得HR，且无明显不良反应；转换治疗前肝功能异常未进一步加重，转换治疗后均逐渐恢复正常。APAG临床长期应用中无明显肝毒性，其安全性相较于其他同类TPO-RA更为优异。

IST联合TPO-RA无效AA患者应尽可能选择合适供者进行异基因造血干细胞移植挽救治疗[22]。TPO-RA难治性AA的药物转化治疗相关研究尚在初始阶段，许多问题尚无肯定回答。我们认为，除TPO-RA不耐受患者可依据药物安全性特征在同类制剂中相互转换外，TPO-RA难治性AA患者的药物转换更应参照其相应的药效学强度，由相对弱作用转换为更强作用制剂。就药效学强度和安全性特征而言，APAG无疑是一较好的转换治疗选择。

我们报告了TPO-RA难治性和TPO-RA不耐受患者的APAG转换治疗结果，提示对于无条件进行HSCT的患者，APAG转换治疗不失为一较好的替代策略。鉴于回顾性研究固有缺陷，患者TPO-RA转换前治疗方案及疾病严重程度等基线特征不均一、缺少相应对照组以及患者样本量较小等，本研究结果难免偏倚，需更大样本前瞻性随机对照研究证实。
